# Extensively Drug-Resistant *Neisseria gonorrhoeae* Strain, Canada

**DOI:** 10.3201/eid3107.250023

**Published:** 2025-07

**Authors:** Brigitte Lefebvre, Irene Martin, Robyn Thorington, Julian Gitelman, Andrew Bui-Nguyen, Claude Fortin, Judith Fafard

**Affiliations:** Laboratoire de santé publique du Québec, Sainte-Anne-de-Bellevue, Canada (B. Lefebvre, J. Fafard); National Microbiology Laboratory, Winnipeg, Manitoba, Canada (I. Martin, R. Thorington); Direction régionale de santé publique, CIUSSS du Centre-Sud-de-l'île-de-Montréal, Montreal, Quebec, Canada (J. Gitelman); Clinique Quorum, Montreal (A. Bui-Nguyen); Centre Hospitalier de l’Université de Montréal, Montreal (C. Fortin); Faculté de médecine de l’Université de Montréal, Montreal (C. Fortin, J. Fafard)

**Keywords:** Neisseria gonorrhoeae, antimicrobial resistance, bacteria, gonorrhea, antimicrobial susceptibility surveillance, ceftriaxone, ceftriaxone-resistant, cefixime, cefixime-resistant, mosaic *penA* allele, *penA*-60, sexually transmitted infections, Canada

## Abstract

We identified a case of extensively drug-resistant (ceftriaxone- and cefixime-resistant and high-level azithromycin-resistant) *Neisseria gonorrhoeae* in Canada. The strain harbors the *penA* 60.001 allele, which includes the A311V and 23S rRNA A2059G single-nucleotide polymorphisms associated with high-level azithromycin resistance. The infection was likely acquired during travel in Cambodia.

*Neisseria gonorrhoeae* is the second most common sexually transmitted infection reported in Canada; the 92.34 cases/100,000 population reported 2022 represents an increase of 146.2% since 2010 (*1*). *N. gonorrhoeae* with third-generation cephalosporin resistance is in the high-priority category on the Bacterial Priority Pathogens List published by the World Health Organization in 2024 (https://iris.who.int/bitstream/handle/10665/376776/9789240093461-eng.pdf). Ceftriaxone is 1 of the last remaining empiric treatments options available for gonorrhea infection and is currently the recommended therapy in Canada. In 2017, we described a ceftriaxone-resistant *N. gonorrhoeae* isolate identified in North America (*2*). In 2018, we reported on the international dissemination of ceftriaxone-resistant *N. gonorrhoeae* isolates (*3*), identified in Japan as FC428. More than 10 extensively drug-resistant (XDR) *N. gonorrhoeae* isolates have been reported worldwide, most since 2022 (*4*). Those XDR isolates were found to harbor the *penA*-60.001 allele associated with ceftriaxone resistance and the 23S rRNA A2059G single-nucleotide polymorphism associated with high-level azithromycin resistance. We report an XDR *N. gonorrhoeae* isolate (no. 69155) identified in Canada that was cefixime and ceftriaxone resistant and has a high level of azithromycin resistance. 

## The Study

In May 2024, a man in his 40s experiencing urethritis sought care in an outpatient STI clinic in Montreal, Quebec, Canada. A strain of XDR *N. gonorrhoeae* from a urethral specimen was isolated at Centre Hospitalier de l’Université de Montréal. The patient reported that he had traveled to Cambodia and had sex with female sex workers there. He reported sexual contact with women only. He denied sexual contacts in Canada in the 60 days before the onset of his symptoms and had no sexual contacts upon his return. He was treated with 1 dose of oral azithromycin (2 g) combined with cefixime (800 mg) in accordance with treatment guidelines for sexually transmitted infections published in 2020 by Institut national d’excellence en santé et en services sociaux (INESSS) (https://www.bibliotheque.assnat.qc.ca/DepotNumerique_v2/AffichageNotice.aspx?idn=101746) but was still symptomatic at the time of the test of cure (TOC) by nucleic acid amplification tests (NAAT) 2 weeks later. We prescribed a second round of treatment with 1 intramuscular dose of ceftriaxone (250 mg) and oral doxycycline (100 mg 2×/d for 7 days); 2 weeks after the completion of the second treatment, the TOC by NAAT still remained positive. Because the patient was still symptomatic, providers used a syndromic approach; he was treated for clinical urethritis in accordance with the INESSS guidelines. The doxycycline was used to treat a possible *C. trachomatis* infection and not the *N. gonorrhoeae* infection. A third treatment of intramuscular ceftriaxone (500 mg) was successful; urethral culture and urine NAAT were negative at the third TOC.

We confirmed the bacterial isolate 69155 to be *N. gonorrhoeae* by matrix-assisted laser desorption/ionization time-of-flight mass spectrometry (bioMérieux, https://www.biomerieux.com) and by whole-genome sequencing. We confirmed antimicrobial susceptibilities by agar dilution in accordance with Clinical and Laboratory Standards Institute (CLSI) guidance (*5*). The ceftriaxone MIC for this *N. gonorrhoeae* isolate was 0.25 mg/L, which is susceptible by CLSI guidelines and resistant by EUCAST guidelines (*6*). The cefixime MIC was 2 mg/L, which is nonsusceptible by CLSI guidelines and resistant by EUCAST guidelines. CLSI does not have a resistant breakpoint for cefixime or ceftriaxone; however, it defines MIC <0.25 mg/L as susceptible. EUCAST defines cefixime and ceftriaxone resistance as MIC >0.125 mg/L, whereas World Health Organization defines decreased susceptibility to cefixime as MIC >0.25 mg/L and to ceftriaxone as MIC >0.125 mg/L (*7*). Although there is no consistent resistance breakpoint, MICs of 0.25 mg/L for ceftriaxone and 2 mg/L for cefixime were previously reported in possible treatment failure cases (*8*,*9*). The isolate was also resistant to azithromycin (MIC >256 mg/L), ciprofloxacin (MIC 4 mg/L), tetracycline (MIC 32 mg/L), and penicillin (MIC >256 mg/L) but susceptible to spectinomycin (MIC 8 mg/L). We also determined MICs for gentamicin (MIC 4 mg/L) and ertapenem (MIC 0.5 mg/L). 

We performed molecular typing in silico from whole-genome sequence data and submitted to the National Center for Biotechnology Information Sequence Read Archive (BioProject PRJNA415047, Sequence Read Archive no. SRR33123384). We sequenced the strain using Illumina NextSeq platform (Illumina, https://www.illumina.com) and used genomic quality, assembly, and annotation pipelines as previously described (*10*). We identified sequence type (ST) 16406 by multilocus sequence typing (MLST), ST-22862 by *N. gonorrhoeae* multiantigen sequence typing (NG-MAST; https://pubmlst.org/organisms/neisseria-spp), and ST-5793 by *N. gonorrhoeae* Sequence Typing for Antimicrobial Resistance (NG-STAR; https://ngstar.canada.ca). The isolate had the mosaic *penA*-60.001 allele associated with ceftriaxone resistance and the A2059G single-nucleotide polymorphism in 23S rRNA associated with high-level azithromycin resistance. The *penA*-60.001 allele contains the A311V amino acid substitution, which is highly associated with ceftriaxone resistance (*11*). The MLST-determined ST16406 and *penA*-60.001 allele of the study isolate in Canada was identical to that of XDR *N. gonorrhoeae* strains isolated in Cambodia, Austria, United Kingdom, and France in 2022–2023 (*4*). Because only 1 strain from initial sampling was available, we did not perform genetic comparisons with other strains from this patient.

To compare the XDR *N. gonorrhoeae* strain we isolated with other ceftriaxone-resistant isolates, we performed phylogenetic analysis to determine single-nucleotide variants (SNV) using the custom Galaxy SNVPhyl20 pipeline (SNVPhyl version 1.0.1b Paired-End, https://github.com/phac-nml/snvphyl-galaxy) and included publicly available, internationally reported ceftriaxone-resistant and XDR gonorrhea genomes (BioProject PRJNA909328) (*12*) (Figure). The phylogenetic tree demonstrates the similarity of the Canada XDR–*N. gonorrhoeae* 69155 isolate to gonorrhea genomes reported in Southeast Asia; the study isolate differs from isolate SRR22570596 by 320 SNVs and SRR22570612 (BioProject PRJNA909328) by 140 SNVs.

**Figure:**
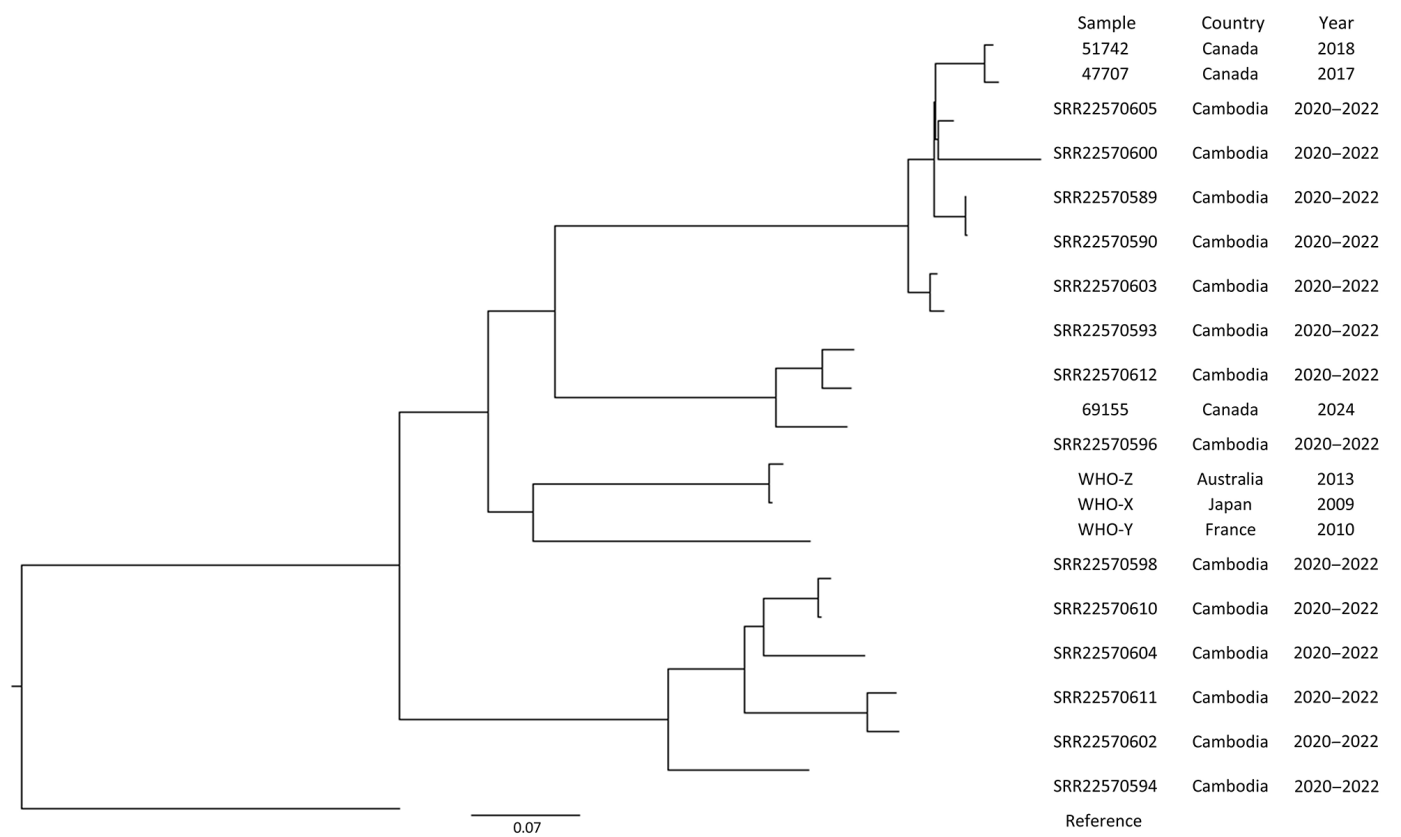
Phylogenetic analysis of *Neisseria gonorrhoeae* isolates in study of extensively drug-resistant *N. gonorrhoeae* infection, Canada. Isolate 69155 was identified from the patient in this investigation. Tree was generated using 3,691 sites. Scale bar indicates number of substitutions per site.

## Conclusions

We identified the bacterial isolate 69155 from a patient in Canada as a ceftriaxone-resistant and high-level azithromycin–resistant *N. gonorrhoeae* strain. The strain is of ST-16406 (MLST) and contains the *penA-60.001* allele identical to cases previously reported in France, United Kingdom, Austria, and Cambodia. The patient’s infection was not cured by cefixime (800 mg) but by a higher dose of ceftriaxone (500 mg). Consistent with US Centers for Disease Control and Prevention recommendations (*13*), Quebec has recommended use of ceftriaxone (500 mg) as first-line treatment since September 2024 (*14*).

In 2020, guidelines in Europe recommended the use of ceftriaxone (1 g) as probabilistic treatment for uncomplicated genital infections (*15*). The European Centre for Disease Prevention and Control noted an increase of antimicrobial resistance in 2022 for *N. gonorrhoeae* (*4*). Moreover, >6 cases of XDR *N. gonorrhoeae* infection have been reported in Europe since 2022. 

The surveillance of antimicrobial resistance in *N. gonorrhoeae* is crucial in the context of treatment failure and the dissemination of XDR strains worldwide. Our isolate was susceptible to ceftriaxone according to CLSI guidelines but resistant according to EUCAST guidelines; we therefore recommend using caution when interpreting borderline MICs. In Quebec, we published a directive asking frontline laboratories to promptly submit all third-generation cephalosporin borderline MIC isolates to the provincial laboratory for testing.
